# Exploring the use of dalfampridine (4‐aminopyridine) for treatment of ataxia in cerebellar stroke

**DOI:** 10.1002/pmrj.70036

**Published:** 2025-10-02

**Authors:** Arjun Kotwal, Prashant Yadav, Veyola Rezkalla, Alison Mautner, Gilbert Siu

**Affiliations:** ^1^ Rowan‐Virtua SOM Stratford NJ USA; ^2^ Inspira Health, Department of Physical Medicine and Rehabilitation Vineland NJ USA; ^3^ Rowan‐Virtua SOM, Department of Physical Medicine and Rehabilitation Stratford NJ USA

A 45‐year‐old man with no known medical history presented to the emergency department with acute onset of muscle weakness and dysarthria. Upon further evaluation, magnetic resonance imaging of the brain revealed an acute infarction of the right cerebellum. Further physical examination showed significant right‐sided ataxic movements, postural instability, and difficulty with activities of daily living. The patient was diagnosed with an acute cerebellar stroke.

After admission to an acute inpatient rehabilitation hospital, the patient began to improve his motor symptoms, regaining significant strength in his right upper and lower limbs. However, he continued to experience significant gait ataxia. After 2 weeks with minimal improvement, the patient consented to a trial of dalfampridine to improve his ataxia.

On day 16 post stroke, 10 mg of dalfampridine twice daily was administered to the patient. Over the next week, he demonstrated significant improvement in his balance, coordination, and ambulation. While walking, the patient no longer leaned toward the left side and improved from fully dependent to requiring moderate assistance for ambulating up to 100 feet. He did not experience any significant adverse effects from the medication and was successfully discharged home. Dalfampridine continued until the patient was followed up with an outpatient neurologist.

Ataxia is a prominent and debilitating symptom of cerebellar strokes.^1^ Persistent poststroke ataxia can cause severe limitations affecting not only activities of daily living but also significant reductions in both long‐term quality of life and poststroke functional recovery. Poststroke rehabilitation is exceptionally taxing on patients, particularly if ambulation is affected.

An intervention that could improve, or eliminate, poststroke ataxia in patients could have a significant impact on their recovery and rehabilitative course. Improved ambulation may provide patients with the motivation needed to continue rehabilitation or serve as a breakthrough for addressing other residual deficits. There are limited pharmacologic agents to help treat ataxia in cerebellar strokes, and most of these medications are rarely successful.

Dalfampridine (also known as 4‐Aminopyridine) is a member of the isomeric pyrimidines drug class and is a relatively new medication approved by the Food and Drug Administration in 2010 for increasing the walking speed of patients with multiple sclerosis. Members of this class are potent antagonists of the Kv1 family of voltage‐gated potassium channels.^2^ Though the mechanism for this effect is not fully understood, it is believed to improve action potential conduction within demyelinated axons by blocking newly exposed internodal potassium channels and preventing them from slowing or weakening the signal.^3^ Additionally, dalfampridine has been shown to act as an agonist of voltage‐gated calcium channels, further improving both central nervous system synaptic and neuromuscular junction function as a result.^2^ This medication has been part of numerous studies that show it is also successful when given “off label” in improving mobility of patients with a wide range of neurological conditions outside of multiple sclerosis.^4^, ^5^ In particular, it has been shown to improve mobility in conditions involving neuromuscular junction dysfunction or axonal demyelination.^2^, ^3^, ^4^

The recommended dosing for clinical usage of dalfampridine is 10 mg twice daily.^3^ It is almost entirely (95.9%) renally excreted, with an elimination half‐life of 5.2–6.5 hours.^5^, ^6^, ^7^ The most common adverse effects reported with usage of dalfampridine include increased risk of urinary tract infections, insomnia, headaches, dizziness, nausea, constipation, and less commonly, seizures and arrhythmias.^5^, ^6^, ^7^

Regarding strokes, only one research article investigated whether dalfampridine improves ambulatory dysfunction after an ischemic stroke. This randomized, double‐blinded study by Page et al. found no significant treatment effect on patient performance on the 2‐minute timed walk test.^8^ However, this study did not specifically investigate cerebellar stroke and excluded any patients who had experienced a stroke within the past 6 months, rather than examining the immediate post‐stroke recovery period. Therefore, to our knowledge, this is the first case reported of dalfampridine improving post‐stroke ataxia within the early stroke recovery stage. Despite early and rapid improvements of upper and lower extremity strength after admission to acute inpatient rehabilitation, our patient continued to struggle with ataxia and postural instability. The patient frequently leaned over to his right side causing difficulty balancing while ambulating. This caused the patient significant distress, and he expressed feeling down and defeated despite the progress he had achieved. Upon starting dalfampridine, the patient corrected his posture within a few days of treatment. With this correction, the patient made significant and rapid gains with ambulation, improving his mood as well.

Dalfampridine may be a useful pharmacologic treatment for patients with cerebellar stroke with persistent ataxia and coordination deficits that do not improve with traditional rehabilitation modalities, especially if given early. To our knowledge, this is the first documented case of dalfampridine utilized in cerebellar stroke recovery to improve gait ataxia. These findings suggest a potential role for dalfampridine in enhancing functional outcomes in cerebellar stroke populations and warrant further investigation.

## FUNDING INFORMATION

There is no funding, grants, or equipment provided for this clinical letter.

## DISCLOSURE

Financial disclosure statements have been obtained, and no conflicts of interest have been reported by the authors or by any individuals in control of the content of this article.
